# The Use of Estrone-3-Glucuronide and Pregnanediol-3-Glucuronide Excretion Rates to Navigate the Continuum of Ovarian Activity

**DOI:** 10.3389/fpubh.2018.00153

**Published:** 2018-05-31

**Authors:** Leonard F. Blackwell, Delwyn G. Cooke, Simon Brown

**Affiliations:** ^1^Institute of Fundamental Sciences, Massey University, Palmerston North, New Zealand; ^2^Science Haven Limited, Palmerston North, New Zealand; ^3^Deviot Institute, Deviot, TAS, Australia; ^4^College of Public Health, Medical and Veterinary Sciences, James Cook University, Douglas, QLD, Australia

**Keywords:** fertility, home urine test, menstrual cycle, estrone glucuronide, pregnanediol glucuronide

## Abstract

The patterns of a woman's normal ovarian activity can take many forms from childhood to menopause. These patterns lie on a continuum ranging from no ovarian activity to a fully fertile ovulatory cycle, but among the other defined patterns are cycles with anovulatory ovarian activity, including luteinized unruptured follicles (LUFs), and ovulatory cycles with deficient or short luteal phases. For any woman, these patterns can occur in any order, and one can merge into the next, without an intervening bleed, or be missed entirely. Consequently, it is not yet possible to predict the pattern of a future cycle, but it is possible to use our knowledge of the continuum to interpret the current cycle, which has clear implications for the management of personal fertility. An individual's position in the continuum can be monitored directly in real time by daily monitoring of ovarian hormone excretion rates, without either calendar-type calculations or reference to population means and standard deviations. The excretion of urinary estrone glucuronide (E1G) gives a direct measure of follicular growth, and the post-ovulatory rise in urinary pregnanediol glucuronide (PdG) following an E1G peak provides good evidence of ovulation. Specific values of the PdG excretion rate can be used to determine whether a cycle is anovulatory with or without a LUF, or is ovulatory and infertile or ovulatory and fertile. These specific values are important signposts for navigating the continuum. For a woman to take advantage of the knowledge of the continuum, the data must be reliable, and their interpretation has to be based on the underlying science and provided in an appropriate form. We discuss the various factors involved in acquiring and providing such information to enable each woman to navigate her own reproductive life.

## Introduction

The achievement of pregnancy requires the precise integration of physiological, biochemical, and anatomical events of a woman's menstrual cycle. These events can be monitored using daily cervical mucus characteristics, the basal body temperature (BBT) and ovarian steroid levels, among other measurements. Observations made each day during the menstrual cycle can be used to generate a profile for an individual woman. When interpreted appropriately, this profile provides a woman with useful information about the events and their timing, and her current fertility status. However, even consecutive cycles from the same woman can vary considerably from month to month. This cycle variation lies within a continuum (1) ranging from an anovular cycle, which happens when one or more of the steps occur(s) inappropriately, to a fertile cycle, which only happens when all the steps occur appropriately within tolerance.

Changes in the levels of the ovarian steroids are directly related to the underlying ovarian physiology because these compounds emanate from the ovary (2). The growth of a follicle is related unambiguously to the rate of secretion of estradiol from the follicle, and ovulation and corpus luteum formation are indicated unambiguously by rising progesterone levels. The corollary of this is that both estrogens and progesterone, or their urinary metabolites estrone-3-glucuronide (E1G), and pregnanediol-3-glucuronide (PdG), respectively, have to be monitored if the cycle is to be understood. All other markers are indirect, in which case they are nonlinearly dependent on the levels of the ovarian steroids and can only be used to *infer* that specific events *might* follow.

If ovarian steroids, or their urinary metabolites, are to be used to monitor the menstrual cycle, at least four criteria have to be fulfilled. First, it must be established that changes in the levels of the steroids are quantitatively or semi-quantitatively related to physiologically significant events. In this case, a woman's minimal requirement is that ovulation and the bounds of the fertile period are reliably identified. Quite reasonably, many women have higher aspirations than this and the continuum provides the basis for such a higher order understanding of the cycle. Second, the assay technique used to monitor the analytes must be shown to be specific, accurate and precise. For an assay to be useful it must measure only the analyte of interest and it must do so with suitable sensitivity and a suitable working range. Third, unless 24-h urine samples are employed, it is necessary to account for the inevitable natural fluctuations in both the total volume of urine in a sample and the time between voids (rate of urine volume production). Fourth, the data obtained during the menstrual cycle have to be interpreted in a coherent fashion. This is especially important because the E1G and PdG profiles of apparently “normally cycling” women can change from one cycle to the next. For a woman monitoring her own fertility, this means that her own data for the current cycle, not an average of her own or other womens' cycles, have to be interpreted. The continuum, as defined by Brown (1), provides a rational basis for interpreting each individual cycle, but its use depends on a complete understanding of the physiology, reliable assays for the ovarian steroids, and correction for the urine production rate.

Our purpose is to provide clinicians with data and reference material to enhance their understanding of the continuum. We have developed guidelines based on many years of experience with measurements of ovarian steroid excretion rates during individual menstrual cycles. Our focus is on monitoring individual cycles in real time. We stress the importance of correcting for urine production rate to do this and demonstrate that such a correction allows the use of universal excretion rate thresholds for pregnanediol (or its glucuronide) in providing signposts to guide passage through the continuum. In doing so we define the various deficient cycle patterns in a clinically practical manner. The understanding of the continuum, and what is required to navigate it, is of fundamental importance for managing infertility.

## Demonstrating the relevance of quantitative measurement of analytes as fertility markers

To establish that measurements of a biomarker are quantitatively or semi-quantitatively related to physiologically significant events is not a simple matter. The first requirement is to establish that the analyte reflects only the physiology of interest. Then it is essential to show that the test is not confounded by extraneous factors such as measurement errors, matrix effects, concentration effects, the (bio-)chemistry of the test or other non-specific effects. Given this, it takes a considerable amount of work to establish that the test is measuring the marker of choice accurately and the underlying activity of the organ correctly.

This was first achieved for ovarian estrogens in the 1950s when a chemical method was developed (3, 4). This assay measured the sum of hydrolysed metabolites of ovarian estradiol excreted in urine (5). Using methods such as isotopic dilution it was established that the assay measured only the urinary metabolites of ovarian estradiol, and that the excretion rate of the sum of estrone, estradiol, and the estriol metabolites is directly related to the ovarian production rate of estradiol (6). It is well established that follicular growth is related to the ovarian estradiol production rate and the urinary excretion rate of estrogens (or E1G) (7–11). An assay for urinary pregnanediol (Pd), a metabolite of progesterone produced in this assay by hydrolysing PdG, was established with similar care (12). It is important to note that both of these assays were applied to 24-h urine collections and the result was expressed as an amount [in micrograms (μg)] per 24 h which is the rate of excretion of the steroid. This is independent of the volume of urine into which the steroid is excreted.

## Characterizing the continuum

Work carried out over many years with careful analysis of many individual cycles, including pregnancy cycles, resulted in the concept of the continuum by Brown (1). This concept arose from finding that there was a progression from anovular activity of childhood, through the establishment of cycling to fully fertile ovular cycles during adolescence and then back again as a woman approached the menopause. These same stages were observed during years of monitoring gonadotrophin therapy at the Royal Women's Hospital in Melbourne during the 1970s and 1980s (13–15). Based on a detailed experimental model (13), each of these cycles could be understood as normal responses to a woman's life and her physiological state. In these cases the hormonal cycle patterns were compared with (i) laparoscopy and ultrasound which show ovulation directly, and (ii) endometrial biopsy which distinguishes between estrogenic and progestogenic control of the development of the endometrium.

The continuum was recognized and defined in terms of the urinary excretion rates of (i) pregnanediol (Pd) or its glucuronide (PdG) and (ii) total estrogens (TE) or estrone glucuronide (E1G). As the parent compounds, progesterone and estradiol, emanate directly from the ovary, unlike luteinising hormone (LH), follicle stimulating hormone (FSH) and the other measures often used to monitor the cycle, Pd(G), TE, and E1G provide a direct link with the events in the ovary (2). The excretion rates of these steroids change characteristically during the menstrual cycle and specific hormonal patterns are associated with particular cycle types.

The key PdG excretion rates are thresholds that generally are exceeded if particular menstrual cycle events have occurred. While it does not really matter whether serum hormones, urinary PdG and E1G or urinary Pd and TE are considered, the thresholds differ and it matters greatly that the serum values are concentrations whereas the urinary values are excretion rates. Experience indicates that the excretion rate of PdG will exceed 13.5 μmol/24 h within 6 days of the estrogen peak day in the luteal phase of a fertile, conceptual (and therefore ovulatory) cycle (1). In an anovulatory cycle, the excretion rate of PdG will not exceed 7 μmol/24 h (16). Between these two thresholds lies a third [9 μmol/24 h (16)] that is biochemical proof of ovulation and hence can be used to distinguish a luteinized unruptured follicle (LUF) from an inadequate luteal phase.

A LUF is a follicle that has been luteinized and has either failed to rupture or has ruptured in such a way that the ovum is not released (17). Given this definition, diagnosis is best made on the basis of ultrasound, although LUFs were originally described based on laparoscopy (18). However, LUFs are also associated with lower (19–22) or more slowly rising (23) progesterone (or Pd or PdG) levels, although there are some exceptions (24). Based on this, we employ a hormonal definition of a LUF based on the threshold PdG excretion rate of 9 μmol/24 h PdG (which is equivalent to the original 2 mg/24 h Pd), as has been accepted for decades, is biochemical proof of ovulation (16). It follows that lower values indicate that ovulation has not occurred. However, the fact that progesterone levels rise above the follicular phase baseline means that some luteinization must have occurred. From a biochemical point of view this is a LUF.

These thresholds have been confirmed over many years by over a million assays and the associated clinical observations obtained during the study of thousands of individual cycles. The only way to demonstrate definitively that a cycle is fertile is that it ends in pregnancy, but it can not be concluded that a cycle is infertile just because it does not. Nevertheless, the threshold we take to indicate fertility (a value in excess of 13.5 μmol/24 h PdG within 6 days of the estrogen peak day) can be examined by taking pregnancy as a proxy for a fertile cycle. Our own data from 213 cycles yield acceptable estimates of the sensitivity (1.00 [95% CI: 0.93, 1.00]), specificity (0.35 [95% CI: 0.28, 0.43]), positive predictive value (PPV, 0.32 [95% CI: 0.30, 0.35]), and negative predictive value (NPV, 1.00) of the threshold. We emphasize that the low specificity and PPV are to be expected because not every potentially fertile cycle results in pregnancy, whereas, the high sensitivity and NPV merely reflect the fact that no pregnancies resulted from those cycles in which the threshold was not exceeded throughout the 6 days following the estrogen peak day. Some further substantiation of the reliability of this threshold can be obtained from the data of Brown et al. (15) who used a slightly lower threshold (equivalent to about 11.2 μmol/24 h PdG). Despite this, their 444 cycles yielded very similar values of the sensitivity (0.94 [95% CI: 0.86, 0.98]), specificity (0.52 [95% CI: 0.47, 0.58]), PPV (0.31 [95% CI: 0.29, 0.34]), and NPV (0.97 [95% CI: 0.94, 0.99]). The most significant difference between these two analyses is that there were five pregnancies using the lower threshold, but no pregnancies when it increased to 13.5 μmol/24 h PdG.

The lowest threshold (the equivalent of 7 μmol/24 h PdG) is widely accepted as a marker for the end of the fertile period [(1, 25–28)]. Similarly, the middle threshold (the equivalent of 9 μmol/24 h PdG) is also widely accepted as biochemical evidence of ovulation [(16, 29–31)]. Nevertheless, we accept that there are few reports in which these two urinary Pd or PdG excretion rate thresholds have been considered using either laparoscopy or ultrasound (32). There is further work to be done.

**Figure 1:**
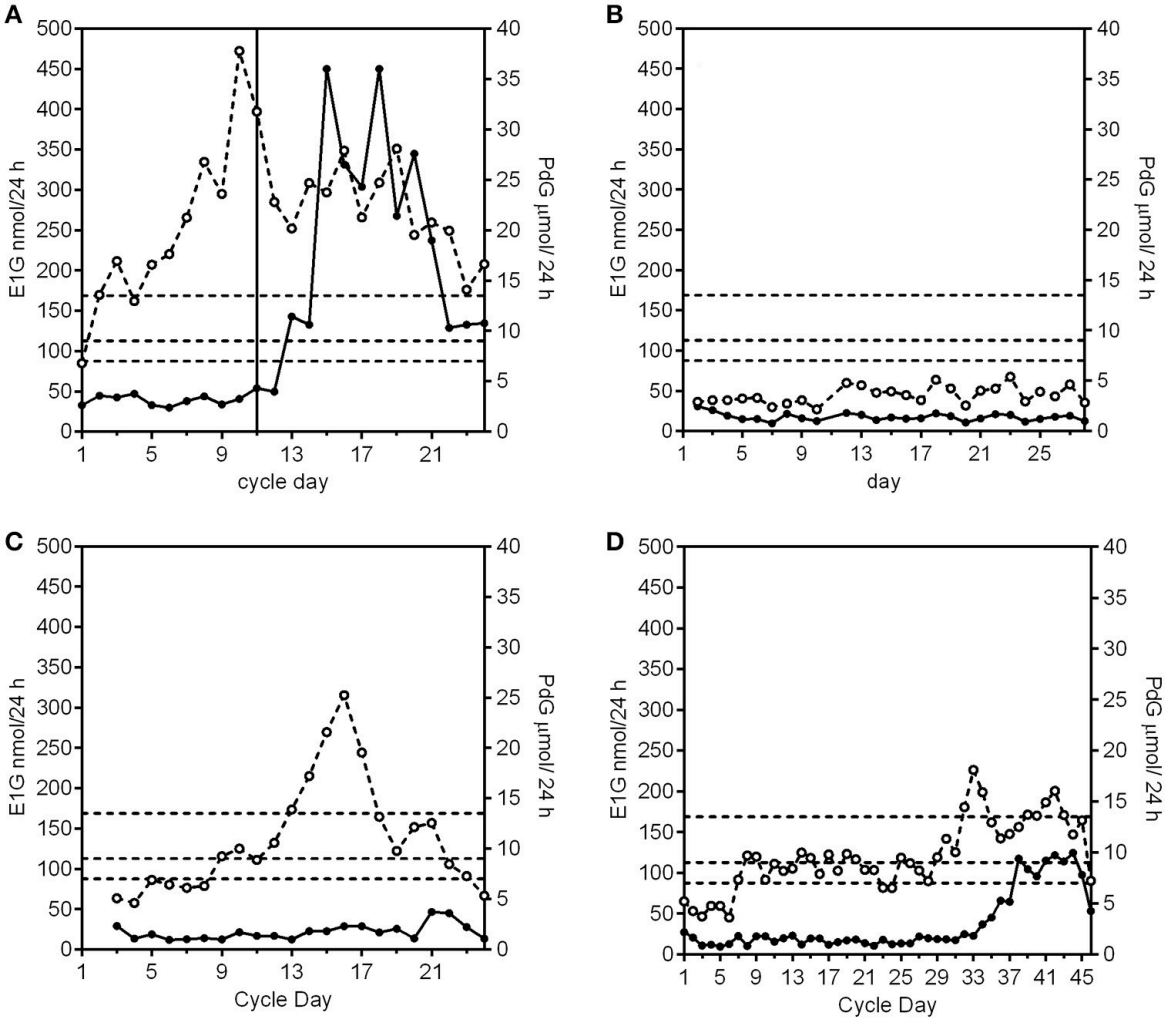
Examples of E1G (°) and PdG (•) excretion rates for an ovulatory, fertile cycle, and three anovulatory cycle subtypes. The PdG thresholds 7, 9, and 13.5 μmol/24 h are shown as broken horizontal lines. **(A)** A normal cycle where the E1G excretion rates fluctuate and the PdG excretion rates exceed all 3 thresholds and the luteal phase length is 14 days. The vertical line is the day of the LH peak. **(B)** An anovular cycle where the E1G excretion rates are consistently low as are the PdG excretion rates. **(C)** An anovular cycle where the E1G excretion rates fluctuate but the PdG excretion rates do not exceed 7 μmol/24 h. **(D)** This cycle has a long anovulatory phase of 28 days where the E1G excretion rates stay effectively constant as do the PdG excretion rates. This was followed by an ovulation, but as the PdG excretion rates did not exceed the 13.5 μmol/24 h threshold the cycle had a deficient luteal phase. Data in this and subsequent figures are derived from (i) the UNDP/UNFPA/WHO/World Bank study on the Hormonal Definition of the Fertile Days of the Cycle by Home Monitoring for Natural Family Planning, protocol #90905 (Ovarian Monitor); (ii) the Fertility Urinary Monitoring Study, approved by the Northern B Health and Disability Ethics Committee, New Zealand, # 12/NTB/18 (collected using the MICT superparamagnetic particle assay); (iii) the Women's Fertility Study, approved by the Central Health and Disability Ethics Committee, New Zealand, # CEN/07/03/014); or (iv) have been published previously (33).

**Figure 2:**
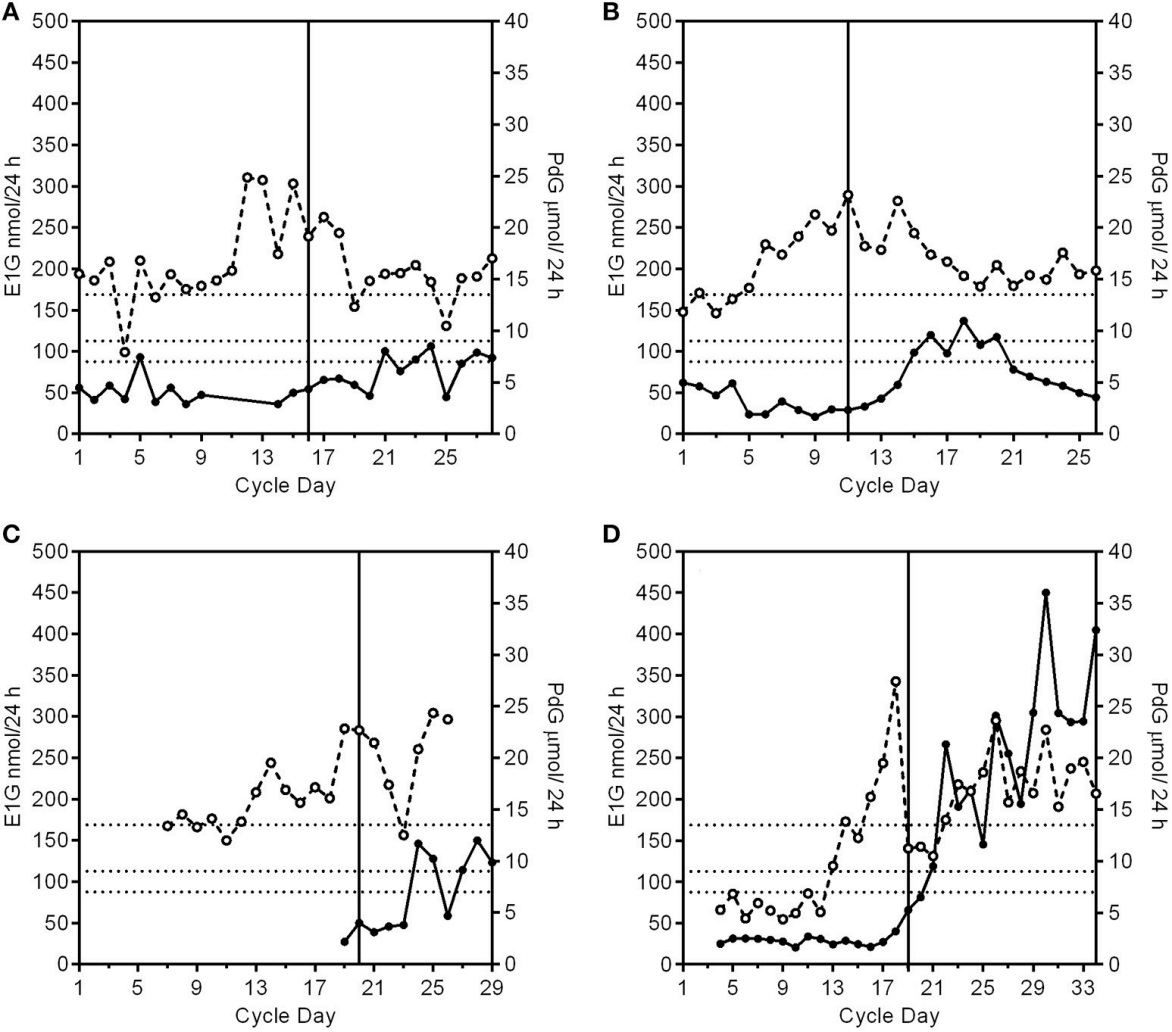
Patterns of PdG production and deficient cycle types. The PdG thresholds 7, 9, and 13.5 μmol/24 h are shown as broken horizontal lines. The vertical line is the day of the LH peak. **(A)** A profile of a cycle with a luteinized unruptured follicle where the E1G excretion rates (°) fluctuate and the PdG excretion rates (•) exceed 7 μmol/24 h, but not 9 μmol/24 h. **(B)** A profile of a cycle with a deficient luteal phase where the E1G excretion rates fluctuate and the PdG excretion rates exceed 9 μmol/24 h, but not 13.5 μmol/24 h. **(C)**. A profile of a cycle with a short and deficient luteal phase. The E1G excretion rates fluctuate and there is an early E1G peak on day 14. The luteal phase was 9 days counted from the peak E1G excretion rate day (day 20) to the day before the next bleed making it a short luteal phase and it was also a deficient luteal phase as defined in **(B)** where the PdG excretion rates exceed 9 μmol/24 h, but not 13.5 μmol/24 h. **(D)** A pregnancy cycle with a classical single E1G rate peak profile in the follicular phase and in which the PdG excretion rates exceed all the thresholds, but the rate has not decreased after 17 days from the E1G peak day.

Nevertheless, the substantial body of work that has been done has yielded the following definitions of distinct cycle types (where the luteal phase is defined as the day after the E1G peak day to the last day of the cycle inclusive):

- A fertile cycle has a luteal phase length of 11–17 days and the PdG excretion rates exceed 13.5 μmol/24 h within 6 days of the E1G peak (Figure 1A).
- Anovulatory cycles
  - In the absence of any follicular growth at all there is no significant systematic change in the E1G or PdG excretion rates (Figure 1B).
  - The E1G excretion rate rises and falls (fluctuates), but the PdG excretion rates do not exceed 7 μmol/24 h (Figure 1C).
  - The E1G excretion rate rises but then stays constant for a period of time and the PdG excretion rates do not exceed 7 μmol/24 h (first 28 days of Figure 1D).
  Anovular cycles can occur in young, lactating and perimenopausal women, elite athletes and any woman experiencing stress. Such cycles are usually sporadic, but are sometimes more frequent.
- Ovulatory or anovulatory cycles in which the E1G excretion rate rises and falls and there are various PdG excretion rates.
  - luteinized unruptured follicle (LUF) cycles show an E1G peak followed by an increase in PdG excretion rates that may exceed 7 μmol/24 h, but do not exceed 9 μmol/24 h (Figure 2A). This is understood to mean a follicle has luteinized but not ruptured. This threshold can be used as a tool for identifying the optimum times for an ultrasound investigation and is an area requiring further research.
  - Deficient luteal phase cycles show an E1G peak followed by an increase in the PdG excretion rates that exceed 9 μmol/24 h, but do not reach 13.5 μmol/24 h (Figure 2B).
  - Short luteal phase cycles show an E1G peak before the rise in the PdG excretion rate which may exceed 13.5 μmol/24 h, but the luteal phase length is 10 days or less (Figure 2C).
- Conceptual cycles are like fertile, non-conceptual cycles in that the PdG excretion rates exceed 13.5 μmol/24 h within 6 days of the E1G peak, but the PdG excretion rates continue to rise past 17 days (Figure 2D) and do not fall again until the pregnancy is over.

It is especially important for any woman monitoring her own fertility to know which of the cycle types in the continuum can be followed by a fertile ovulation *without an intervening menstrual bleed* [for an example see Figure 9 in Brown (1)]. The PdG excretion rate is the key hormonal parameter that can be used to determine what might follow. A PdG excretion rate in excess of 9 μmol/24 h is usually followed by a normal menstrual bleed up to 17 days later. After that any cycle type from the continuum can follow in the subsequent cycle. However, if the PdG excretion rates do not reach 7 μmol/24 h, there are many more possibilities. For example, a completely anovular period (Figure 1B) may be followed, without an intervening bleed, by a normal ovulatory cycle (Figure 1A) or by any of the other possibilities of the continuum. Such a completely anovular period does not help a woman monitoring her own cycle to predict what will follow, so continued vigilance is needed, and she should continue to monitor her cycle. On the other hand, (i) raised estrogen levels followed by a fall, indicating atresia of a developing follicle, can be followed by a withdrawal bleed (Figure 1C), and (ii) raised estrogens (Figure 1D) may result in a breakthrough bleed if the levels are elevated for many days.

## Urinary excretion rates vs. concentrations

To take advantage of the continuum in monitoring the menstrual cycle it is essential that a very simple point is clear: the data must be reliable and quantitative. There is a tendency to believe that any method that yields a number is quantitative. However, just getting a number is not helpful; the numbers must be meaningful, accurate and precise and of the right sort, especially when considering the first rise in E1G levels to mark the beginning of the fertile window (34) and the PdG (and Pd) thresholds. It also needs to be stressed that the measurement is meaningful and reliable only if it is an excretion rate, that is in units of time e.g. μmol/24 h. The concentration of urinary PdG (or Pd) in μmol/L is physiologically meaningless because differences in the volume of the urine sample will affect the concentration profoundly (35) and they cannot be anticipated. Just drinking more or less will affect the PdG concentration, but it will not affect the PdG excretion rate.

One way of thinking of this is to consider that the estradiol secreted by the follicle is analogous to the production of widgets by a factory: it makes sense to express the output of the factory as widgets made per day, but it is less reasonable to express it as widgets per volume of the boxes in which they are packed. In the latter case the apparent productivity of the factory would change (downwards) if widgets were suddenly packed in bigger boxes, but irrespective of the size of the boxes, the number of widgets made per day is a value that the factory manager would understand and find useful. The widget is analogous to a quantity of urinary steroid and the volume of the boxes is analogous to the volume of urine passed when a sample is obtained for testing. The production of ovarian estradiol and progesterone and their subsequent metabolism and excretion are all dynamic processes: they change with time. Excretion rates are amounts per time, whereas concentrations are amounts per volume and the hormone output of the ovary is kinetically related to the former; the concentration is an unreliable approximation.

The original chemical methods of Brown and his contemporaries were all based on complete 24-h urine sample collections, so that variations in the volume of urine excreted were not a factor and the daily variation did not degrade the information being collected. This applies particularly to the PdG assays on which the thresholds used to define the cycle types are based. Pregnanediol glucuronide (or its hydrolysis product, Pd) is the major metabolite of ovarian progesterone. Although there are variations between women, all women produce PdG as their major metabolite and it has proved possible to establish thresholds for Pd and PdG (36). This may not be possible for the urinary metabolites of estradiol as it has long been known that there are considerable variations between women in the production of urinary estradiol, estrone, and estriol glucuronides, and in the ratios in which they are produced (37, 38). For this reason, we specify no E1G thresholds, although some have tried (39). However, a significant increase from baseline in the E1G excretion rate can be used as a marker for the beginning of the fertile window (40).

While giving the excretion rate accurately, 24-h urine collections are not suitable for daily monitoring of individuals in the clinic or at home. Two main approaches have been used to avoid 24 h collections.

The first, proposed by the World Health Organisation (WHO) in the 1980s (41), is to ignore the fluctuations in the volume of urine and *assume* that an overnight, or early morning, urine collection is approximately constant in volume. Though an overnight collection may be more constant than a day time sample, they are still highly variable. Equally concerning is the fact that the distributions of overnight void volumes and urine production rates are positively skewed from which it can be inferred that extreme values are more likely than would be expected were the distribution normal.

The second approach to avoid a 24-h urine collection is to use a sample of at least 3 h duration and then dilute the urine samples to a constant urine production rate which eliminates the effect of fluctuations in void volume (42). We have shown (42) that diluting all urine samples to 150 mL/h before analysis (and then expressing the output as the equivalent μmol PdG/24 h or nmol E1G/24 h) gives the same excretion rates as those obtained with 24 h collections within the experimental errors of both methods (43). There will always be uncertainty about the significance of any measurement of urinary analyte concentration and only the second approach gives the excretion rate of the metabolite.

In an individual, the serum estradiol concentration is usually proportional to the urinary excretion rate of estrone glucuronide (E1G) (38, 44–47). The latter is the product of the urinary concentration of E1G and the urine production rate (in mL/h) and we have shown that it is almost as important to measure the urine production rate as it is to measure the E1G concentration itself. It is essential that fluctuations in urine production rate are taken into account if urinary glucuronides are to be used to monitor the menstrual cycle accurately at home. This is particularly important in the delineation of the limits of the fertile window.

Urinary excretion rates are rarely used in monitoring fertility (1, 36, 37, 48, 49). More often data are reported as urinary analyte concentration normalized to either urinary creatinine concentration or the specific gravity of the urine (50). Recently, there have been several reports based only on urinary analyte concentrations (39, 51–59). While it is likely that creatinine corrected concentrations are less reliable than excretion rates (60, 61), we believe that this is a much better approach than merely relying on the concentration. There are, however, two practical difficulties with creatinine correction which remain. First, a creatinine assay suitable for use by a woman monitoring PdG and E1G in her own home is required in addition to the steroid assays (and the creatinine assay has to be of the same quality as those for PdG and E1G). Second, the values of creatinine-corrected PdG concentration corresponding to the PdG thresholds on which the continuum-based interpretation of the menstrual cycle relies have to be re-established using the alternative combined PdG-creatinine assay system (62).

## The ovarian monitor PdG assays and beyond

We have developed several PdG assays with the potential for home use. Of these, the Ovarian Monitor has allowed women to understand their reproductive lives using the continuum (63) and to identify accurately the precise end of their fertile window (36). Extensive use was made of it for this purpose with home testing in Victoria, Australia, in the 1990s (63). The details of what has to be achieved to develop a suitable assay for PdG are given by Binnie et al. (64). Essentially, the assay must give quantitative and accurate excretion rates for a population of women, these must correlate well with the reference chemical Pd assay on 24-h urine collections (12) and give normal ranges which superimpose on those given in Brown (1). This has been demonstrated for the Ovarian Monitor PdG assay (34). The excretion rates of PdG, measured using the Ovarian Monitor, and Pd, measured using the reference chemical assay, are linearly related [PdG = 3.8 × Pd + 1.0 μmol/24 h, *r* = 0.95, *n* = 227 (65)], and the Ovarian Monitor gives the same excretion rates for PdG (1, 65) as the Pd reference assay after conversion of the Pd reference data from mg/24 h to μmol/24 h. For these reasons we now use the Ovarian Monitor PdG assay as our reference PdG assay because the original Pd assay is not currently available. The PdG enzyme linked immunosorbent assay gives the same excretion rate data as the Ovarian Monitor and thus provides a secondary reference PdG assay (64).

**Figure 3:**
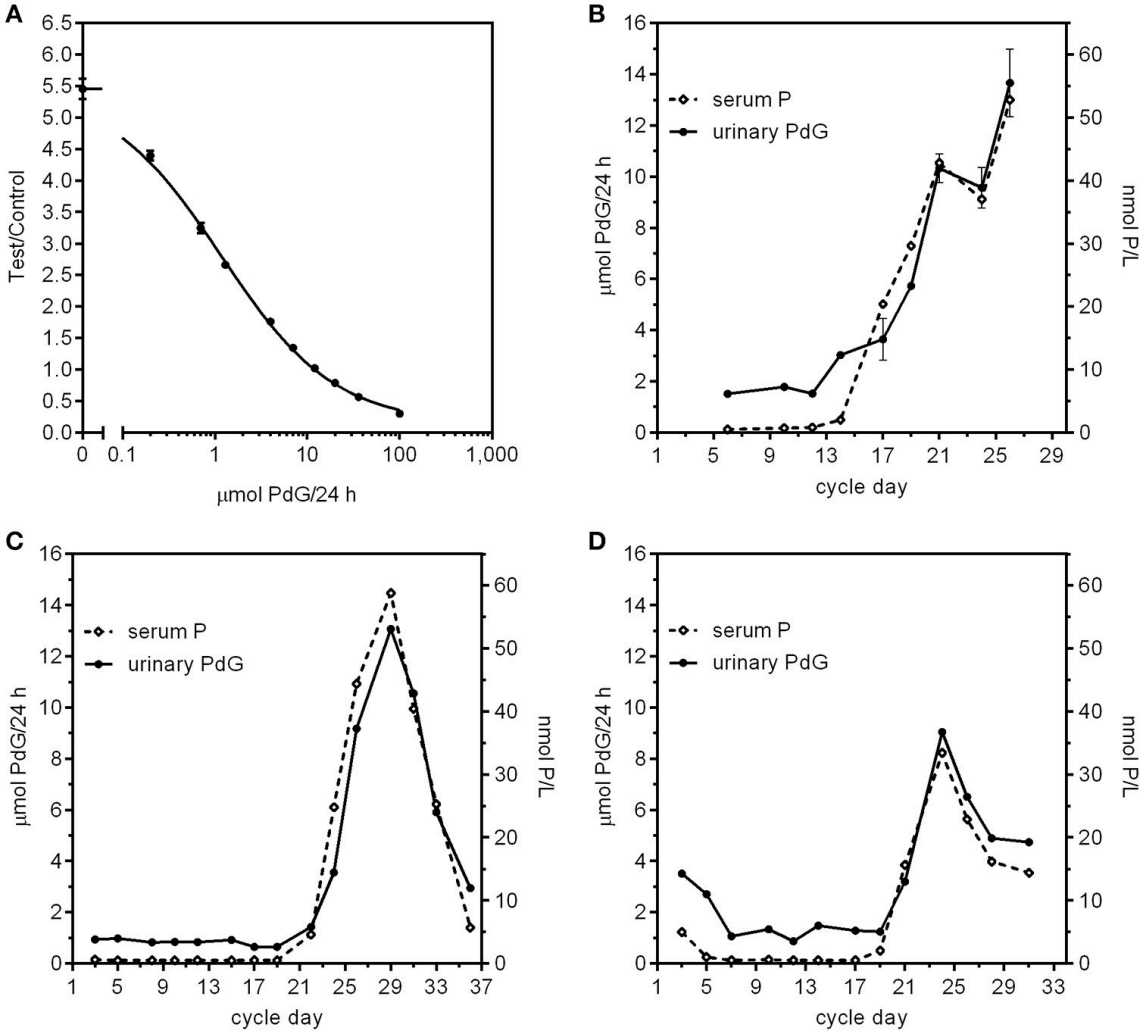
**(A)** PdG standard curve using the MICT superparamagnetic particle assay. The Y axis is the ratio of the magnetic intensity of the particles bound to the test line divided by that of the control particles bound at the control line on the strips. Each point represents the mean (error bars ± SD) of 12 replicates (2 readers, 6 replicates each reader). **(B–D)** Comparison of menstrual cycle profiles from three women using paired serum progesterone concentration and urinary PdG excretion rates. The serum values were obtained using a standard pathology progesterone assay of samples and the urine values were obtained using urine sample collected on the same day diluted to 150 mL/h using the MICT superparamagnetic particle assay and the standard curve in **(A)**. For subject B, the urines were all overnight urine collections and the bloods were all collected later between 10:45 a.m.−1:10 p.m. For subject **(C)**, all the urines were overnight collections and the bloods were all collected later between 9 a.m. and 2:20 p.m. For subject **(D)**, the urines were a mix of overnight and day time urines, but the bloods were all collected later between 9:30 a.m. and 3:50 p.m.

**Figure 4:**
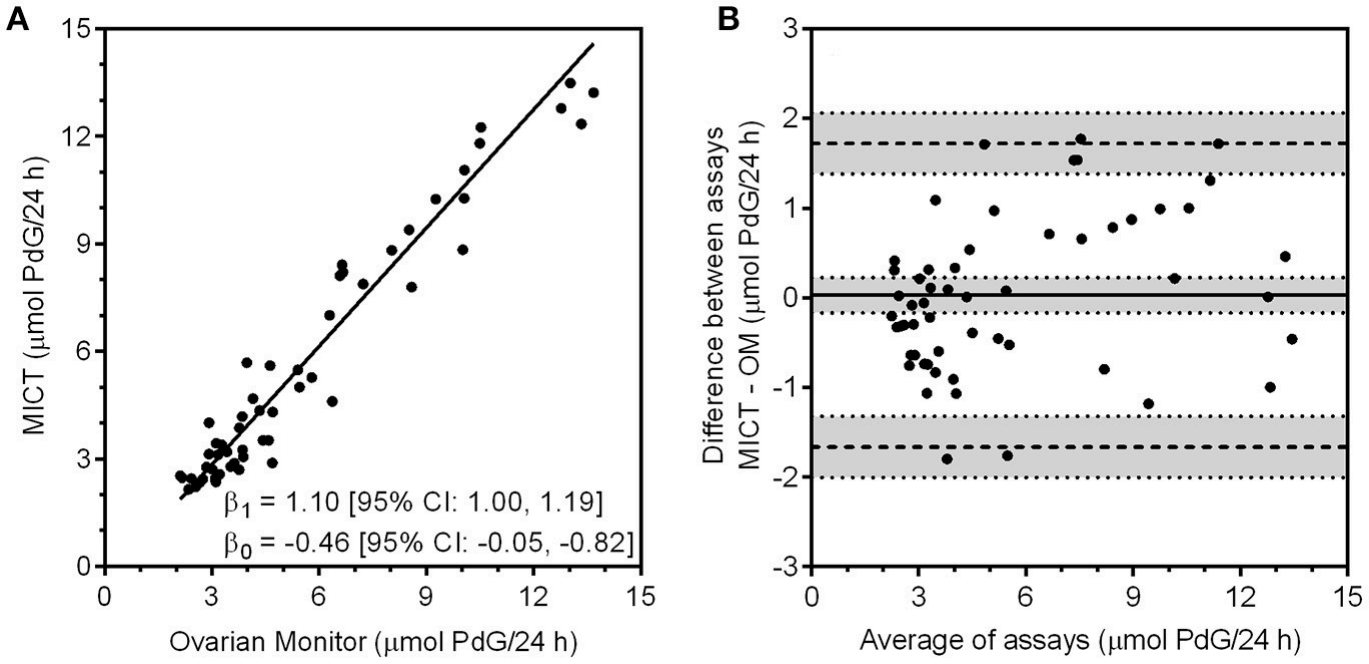
**(A)** Correlation of the PdG values obtained with the MICT PdG assay vs. the excretion rates obtained for the same urine samples with the Ovarian Monitor PdG assay. The upper and lower limits of quantitation were calculated for both assays and only data that were within the range for each assay were correlated. The line shown was obtained by Deming regression. **(B)** The Bland-Altman plot of the data is shown where the difference between the two assays (MICT—OM) is calculated and plotted against the mean of the two values. (68).

Recently we have tested a new superparamagnetic particle assay for PdG using the criteria given above. This method has been validated against the Ovarian Monitor PdG assay and also by comparison of the urinary PdG excretion rates for menstrual cycle samples with the corresponding serum progesterone levels determined in a pathology laboratory. When correlating methods, it is important to be aware of the lowest level of quantitation (LLOQ) of the different methods, and correlate only those values within the working ranges of the assays being compared. The new superparamagnetic particle assay we used here (Figures 1B, 3A–D, 4A,B) employs magnetic immunochemical test (MICT) technology (66). This is based on the measurement of the magnetic field of paramagnetic beads bound in a lateral flow immunoassay. The advantage is that sensitivity of the detector yields both a very low LLOQ and a very substantial working range. We have used superparamagnetic particle assays for both E1G and PdG. Both assays outperform the corresponding serum pathology laboratory assays in that they measure changes in excretion rate for cycle days for which the serum concentrations appear to be at (or below) the LLOQ of the assay. The standard curve for the PdG assay is shown in Figure 3A which confirms its very high precision and remarkable working range. The coefficient of variation for repeated determinations of the excretion rates on the standard curve was < 5% (average 3.5%). This high precision is important as in general the coefficient of variation of the excretion rates derived from the standard curve is approximately double the coefficient of variation of the raw data. In the case of the MICT assays, the parameter is the ratio of magnetic reading of the test and control lines (67).

The Ovarian Monitor PdG assays were designed specifically to measure the key changes in the menstrual cycle (65) and this must be taken into account in inter-assay comparisons. The reactions of the Ovarian Monitor PdG assays are carried out in a single tube using only mildly diluted urine samples (to 150 mL/h of urine collection, which usually corresponds to a dilution by a factor of between 3 and 8). This has two important consequences. First, as with many immunoassays, there is a matrix effect that can lead to spurious results if not taken into account (69). This is minimized in the Ovarian Monitor assays by a pre-incubation step (43, 65). The matrix effect can be diluted out to some extent, but large dilutions of the sample are not possible for home assays of ovarian steroids. Second, the working range of the Ovarian Monitor PdG assay is restricted which means that higher excretion rates can only be measured accurately with further dilution of the samples. Despite these difficulties, there is good agreement between the Ovarian Monitor excretion rates for PdG and the corresponding superparamagnetic assay PdG excretion rates as shown in Figure 4A. There is little bias and no systematic variation in the differences between the two assays (Figure 4B). The relationship between the MICT and Ovarian Monitor (OM) assays is MICT = (1.1 ± 0.1) × OM − (0.46 ± 0.41) μmol/24 h (± 1.96SE) which shows that the assays give the same excretion rates within experimental uncertainty. This implies that the same PdG thresholds (34) might be used with the new superparamagnetic particle assays. The urinary PdG MICT and serum progesterone profiles for the cycles shown in Figures 3B–D also overlap well. However, the working range of the standard pathology laboratory progesterone assay does not cover the very lowest progesterone concentrations (the LLOQ was 0.5 nmol/L), whereas the working range of the superparamagnetic particle PdG urinary assay does cover the lowest PdG excretion rates. For this reason, the cycle data in Figures 3B–D can only be compared on those days when the serum progesterone concentration exceeds about 0.5 nmol/L. During this period, it is clear that there may be a slight delay between the serum concentration and the urinary excretion rate, consistent with the time difference between the midpoint of the overnight urine collection (≈3 AM) and the day time blood collection (≈ 9 AM), and also the time required for progesterone to be metabolized to PdG and excreted. If this is considered, the agreement between the profiles is outstanding and further validates a new superparamagnetic particle urinary PdG assay.

Another important consideration is the antibody. The antibody used in the assays matters and always should be thoroughly characterized (70). In an immunoassay, a signal will almost always be obtained, but great care has to be taken to show that it can be converted into a number which measures the actual amount of analyte present in the sample. No antibody is completely specific for a particular analyte in the presence of high concentrations of a structurally related contaminant. The new PdG assays using urine samples diluted according to time give excretion rates entirely comparable with the Pd chemical assay which is the foundation of the continuum.

## The continuum and cycle averaging

The ovarian steroid profiles of individual cycles have a low signal:noise ratio if the data are not corrected for urine production rate. The predominant response to this in the literature has been to align all cycles relative to a mid-cycle marker, such as the LH peak or the ultrasound derived day of ovulation, and then to calculate daily averages of the data from many cycles. Inevitably, this gives smoother profiles, but it conceals information that is important to the management of a woman's fertility (71).

We are focussed on devices that enable a woman to (i) monitor her own fertility in her own home each day and (ii) integrate the hormone excretion rates and her natural signs of fertility. In particular, we want to define the limits of the fertile window with accuracy and hence to reduce the potential period of abstinence for pregnancy avoidance to the true minimum. At present, a woman has access only to her own cycles and it is probably pragmatic to assume that she has access only to her current cycle. Even if average cycles are available, averages of cycle populations or even a woman's own past cycles are not helpful to an individual dealing with her current cycle. Simply averaging a large number of cycles is meaningless unless they are all the same type of cycle, a problem that the continuum could be helpful in resolving. The only useful aspect of averaged cycles for a woman is the uncertainty envelope within which she might expect her profile to lie, in at least some circumstances, but this still doesn't give her any absolute information about her current cycle. Each cycle reflects a woman's own unique physiological, psychological and environmental circumstances which do not remain static, so her cycle can easily change from month to month. A woman who had an “average” cycle 1 month might well have quite a different cycle the next.

The E1G and PdG excretion rate profiles of an individual provide reliable data which she can use to locate her changing position on the continuum, as well as understand her current cycle as it proceeds. Eventually, her personal interpretation might be enhanced, as suggested previously (35) by integration of other data and data sharing. However, this has to be subject to ethical approval and some semblance of agreement as to some details of measurement, such as correction of urinary measurements for fluctuations in urine production rate (67).

While considering a single cycle may be irrelevant in population research, it is the only case of importance for a woman. Given this, and especially the logical inconsistencies inherent in most cycle averages, we have always analyzed individual cycles using excretion rates. The analysis of over a million estrogen and pregnanediol excretion rates formed the basis of Brown's continuum (1). However, the reluctance to correct for urine production rate has been one of the main impediments to the recognition of the continuum. Women, couples and clinicians require a guide to the intricacies of the menstrual cycle that does not rely on complex rules and minimizes excess abstinence and possible misdiagnoses. The continuum provides such a guide.

With current technological advances, we expect remote cycle monitoring through a device such as a smart phone will become the new reality. This raises many issues, some of which we have considered elsewhere (35). When this point is reached the software might well have access to more of a woman's own data and more sophisticated analyses may well be helpful. Nevertheless, because of the range of cycle variations even in an individual it is unlikely that this will be a simple statistical problem. An example of the variation that can be observed is illustrated clearly in section What can you tell using the continuum?

## What can you tell using the continuum?

Based on the cycle types we have described (section Characterizing the continuum), the PdG and E1G excretion rates enable women to identify the type of their cycle and its place in the continuum and, to some extent, the likely sequelae (1). It is also possible to identify the start and end of the fertile period accurately which gives a shorter fertile window than any other method (34), and thus, depending on the woman's intentions and the nature of her cycle, can assist her to conceive or avoid pregnancy. Daily testing is not necessary as an understanding of the continuum cycle types and their possible sequelae can be used to guide a woman as to the appropriate days for testing. The continuum is also helpful to the many women who have difficulty conceiving, and to their clinicians, because it provides (i) insight into the nature of the problem and its persistence, (ii) a means of assessing whether intervention, such as treatment with clomiphene, is warranted, and (iii) could be used to assist in interpreting events in infertility treatments, for example in clearly identifying the most appropriate day for an ultrasound scan or in helping titrate the dose of gonadotrophins (15).

One example of the continuum as a guide to the sequence of measurements women should make is the WHO sponsored trial of the Ovarian Monitor (72). In that study each woman contributed 6 cycles of data obtained at home with the Ovarian Monitor. During the first two cycles, women measured their E1G and PdG excretion rates daily (32, 34, 72, 73). For the following 4 cycles they monitored their daily E1G excretion rates until a mid-cycle E1G peak was identified and then tested for PdG until the value of 7 μmol PdG/24 h. At this point most, but not all, women stopped collecting data in that cycle (a PdG excretion rate of 7 μmol/24 h indicates the end of the current fertile period and ultrasound measurements of the endometrium at this level confirm that bleeding will follow). The 75 women in the trial contributed 357 cycles in total and 222 of these were cycles 3–6. As the protocol did not require that they continue testing once it had been established that the fertile period had ended, it was possible to establish the cycle type for only 109 cycles. In these 109 cycles, when the PdG excretion rates did not rise rapidly or did not exceed 9 μmol/24 h, the women often continued the measurements long enough into their luteal phases for the cycle type to be identified.

**Figure 5:**
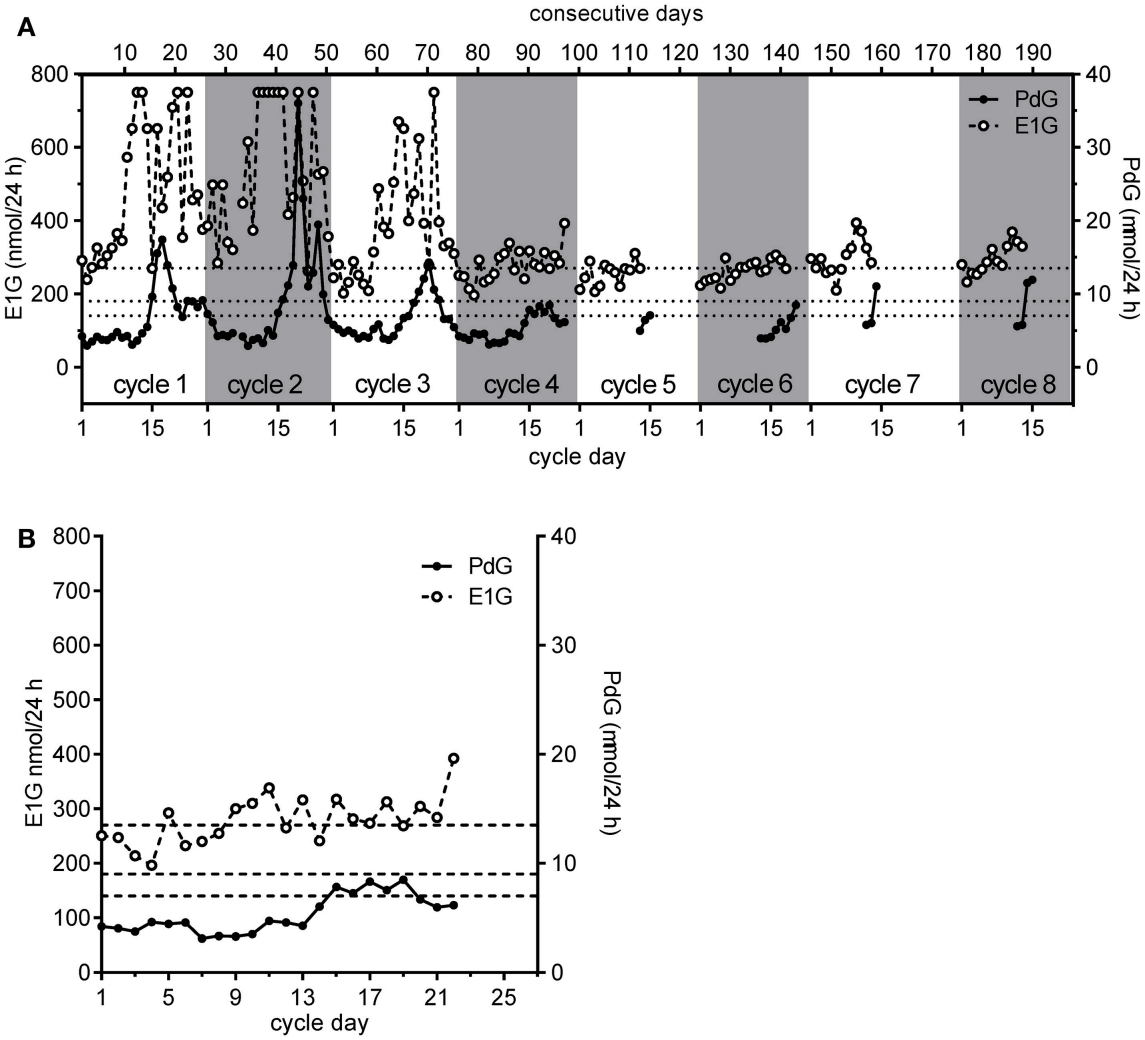
The eight consecutive cycles obtained by a single participant in the WHO study showing her personal continuum **(A,B)** a detailed view of cycle 4 shown in **(A)**. The PdG thresholds 7, 9, and 13.5 μmol/24 h are shown as broken horizontal lines. Further discussion is given in the text.

An example of a woman exhibiting a range of different cycle types is shown in Figure 5A. For this woman, the first two cycles were “normal” as the PdG excretion rates exceeded 13.5 μmol/24 h within 6 days of the E1G peak (1) and she had very high peak day E1G excretion rates - ideally, she should have done an extra dilution of these days for cycles 1 and 2 and repeated them as they were off scale and gave her a truncated E1G peak. For her third cycle, although the E1G excretion rates were again high, the PdG excretion rates suggested a deficient luteal phase. In her fourth cycle, the woman estimated the start of her fertile period on the basis of her mucus symptom as cycle day 7, the mucus peak day as day 13 and the BBT shift day as day 16 (Figure 5B). The E1G excretion rate declined over the first few days of the cycle and the first rise in E1G excretion rate, the start of the potentially fertile phase, was identified by the woman as day 7 (ignoring the spike on day 5). This agrees with the mucus symptom she reported, but the day of E1G peak excretion was day 11 compared with the reported mucus peak day of 13. The last day of fertility, day 14, was the day before the PdG excretion rate first exceeded 7 μmol/24 h and the fertile window was 8 days (days 7–14). However, as the PdG excretion rate did not exceed 9 μmol/24 h the cycle was probably a LUF. The BBT shift day (last day of fertility = BBT + 2) and the cervical mucus symptom (last day of fertility = mucus peak day + 3) indicated to the woman that her infertile period could be assumed to have begun on day 19 and 17, respectively.

The E1G and PdG excretion rate data assisted this woman in at least four ways. First, the period of abstinence was reduced from 12 days (days 7–18), based on the most conservative symptothermal end of potential fertility marker, to 8 days. Second, the cycle was identified as probably involving a luteinized unruptured follicle since the PdG excretion rate exceeded 7 μmol/24 h, but did not exceed 9 μmol/24 h. We remind the reader that this hormonal definition of a LUF differs from that based on observation using ultrasound. Third, the analysis had greater certainty than would have been the case based on other measures, allowing her to live her life with fewer constraints. Fourth and more importantly, she would know from the continuum that this LUF would not be followed by another follicle without an intervening bleed. In practice, she did bleed again on cycle day 25 without any further follicle developing. In the next cycle, cycle 5 she bled heavily for the first 3 days and then had 2 days of spotting including blood stained mucus on day 6. She correctly identified this as the start of potential fertility in this cycle since the protocol required the participants to identify this day by the first day of mucus. In agreement with her mucus symptoms, the E1G excretion rate also rose on cycle day 6. She did not continue her PdG testing long enough into the luteal phase of this cycle to determine the cycle type. However, the next cycle she monitored, cycle 6, had a short luteal phase. She also monitored two more successive cycles not required by the protocol. The seventh cycle was probably normal, but insufficient PdG data were collected to be certain, and this was also the case for the eighth cycle. The E1G excretion rates were considerably lower for the last five cycles than for the first two. However, the last two cycles had greater E1G excretion rates than cycles five and six, perhaps indicating a return to more “normal” cycling.

It is notable, but not especially remarkable, that in only eight consecutive cycles this woman exhibited four different cycle types, and these were clearly identified using. the continuum as (in order): “normal;” “normal;” deficient luteal phase; LUF; undetermined; a short luteal phase, undetermined and undetermined. The three undetermined cycles were undetermined only because measurement of luteal phase PdG excretion rates was stopped too soon. By all standard criteria, this woman would be considered to be cycling “normally” (34), but her very different cycles illustrate one of the problems of averaging the cycles of such “normally” cycling women. If the various cycle types are not distinguished, it is inappropriate to average them, and the corollary is that means and standard deviations can be highly misleading if based on samples that are not comparable.

## Conclusions

Our purpose has been to provide clinicians and others with references and discussion of the key factors involved in the use of the continuum by individual women who hope to avoid or achieve conception. We understand that the information in this review is of little use to women unless they have access to a device which allows them to determine their results at home. Of course, if laboratories correct for urine volume by time dilution and carry out the tests described here, standard validated assays for E1G and PdG should yield data that can be interpreted using the continuum and the PdG thresholds. We would be happy to confirm this with our reference Ovarian Monitor PdG assay. Women deserve a solution which they can rely upon.

The Monitor assay became moribund with the death of Professor James Brown. However, we have now recovered the production of the monitors and tubes at least for research use, and we are also producing prototypes for new, rapid, accurate assays for PdG for clinic based, and home use. In parallel, we are investigating procedures to determine the production rate of a urine sample without any need for information on the time or volume of the collection which will make urine the medium of choice. At present, we do not really contemplate the possibility of serum assays because of the invasiveness and cost of sample collection and the difficulties of sample collection in the home environment. Nor do we do seriously contemplate the use of any other bodily fluids. We believe that our new assays, combined with the new method of determining the urine volume production rate, will allow women to navigate their personal path through the continuum with ease.

## Author contributions

All authors listed have made a substantial, direct and intellectual contribution to the work, and approved it for publication.

### Conflict of interest statement

DC is currently employed by Science Haven Limited, LB is a contractor to Science Haven Limited and SB has been a consultant.
